# Mechanical strain disrupts primary cilia structure and modulates hedgehog signalling in adult chondrocytes

**DOI:** 10.1186/2046-2530-1-S1-P53

**Published:** 2012-11-16

**Authors:** CL Thompson, JP Chapple, MM Knight

**Affiliations:** 1 Queen Mary University of London, UK; 2 Barts and the London School of Medicine and Dentistry, UK

## 

Chondrocytes are the unique cellular component of articular cartilage, the connective tissue covering the converging bone surfaces in joints. Chondrocytes within articular cartilage possess primary cilia although their function is unclear. In osteoarthritis hedgehog signalling, which takes place on the cilium, is aberrantly activated promoting cartilage degradation through the upregulation of ADAMTS-5. The mechanisms for this, and the associated increase in cilia length and prevalence, are unknown. This study tests the hypothesis that alterations in mechanical loading influence cilia length and hedgehog signalling leading to increased ADAMTS-5 expression. Mature bovine articular chondrocytes were subjected to 5%, 10% or 20% cyclic tensile strain (CTS). CTS significantly reduced mean primary cilia length (p<0.05) in a dose-dependent manner such that cilia became progressively shorter with increasing strain magnitude. Indian Hedgehog gene expression was significantly increased by CTS at all strains (p<0.05). Pathway activation (Patched1 gene expression), was observed at 5% and 10% strain but not 20% strain where the greatest reductions in cilia length occurred. Similarly, the mRNA levels of ADAMTS-5 were significantly increased by CTS at 5% and 10% strain (p<0.05) but not at 20% strain. These data suggest that mechanical loading activates hedgehog signalling in adult chondrocytes promoting cartilage degradation and highlights a link between primary cilia structure and function in cartilage disease.

